# Simultaneous Optical Recording in Multiple Cells by Digital Holographic Microscopy of Chloride Current Associated to Activation of the Ligand-Gated Chloride Channel GABA_A_ Receptor

**DOI:** 10.1371/journal.pone.0051041

**Published:** 2012-12-07

**Authors:** Pascal Jourdain, Daniel Boss, Benjamin Rappaz, Corinne Moratal, Maria-Clemencia Hernandez, Christian Depeursinge, Pierre Julius Magistretti, Pierre Marquet

**Affiliations:** 1 Brain and Mind Institute, Ecole Polytechnique Fédérale de Lausanne, Lausanne, Switzerland; 2 Hoffmann-La Roche, Basel, Switzerland; 3 Institute of Applied Optics, Ecole Polytechnique Fédérale de Lausanne, Lausanne, Switzerland; 4 Center for Psychiatric Neuroscience Department, Lausanne University Hospital, Prilly, Switzerland; Universidad de Castilla-La Mancha, Spain

## Abstract

Chloride channels represent a group of targets for major clinical indications. However, molecular screening for chloride channel modulators has proven to be difficult and time-consuming as approaches essentially rely on the use of fluorescent dyes or invasive patch-clamp techniques which do not lend themselves to the screening of large sets of compounds. To address this problem, we have developed a non-invasive optical method, based on digital holographic microcopy (DHM), allowing monitoring of ion channel activity without using any electrode or fluorescent dye. To illustrate this approach, GABA_A_ mediated chloride currents have been monitored with DHM. Practically, we show that DHM can non-invasively provide the quantitative determination of transmembrane chloride fluxes mediated by the activation of chloride channels associated with GABA_A_ receptors. Indeed through an original algorithm, chloride currents elicited by application of appropriate agonists of the GABA_A_ receptor can be derived from the quantitative phase signal recorded with DHM. Finally, chloride currents can be determined and pharmacologically characterized non-invasively simultaneously on a large cellular sampling by DHM.

## Introduction

Regulated and selective transport of ions mediated by ion channels underlies various fundamental cellular processes. Ionic channels represent an important group of therapeutic targets which are modulated by a range of currently prescribed drugs. In particular, chloride channels are involved in several physiological functions such as cell volume regulation, transmembrane fluid transport, muscular activity and neuroexcitability (for review see [1]). Their dysfunction is observed in over a dozen human pathological conditions, including several that affect the nervous system such as epilepsy and certain psychiatric diseases (for review, see [2], [3]). The development of drugs targeted to these chloride conductances represents an important field for developing novel pharmaceutical agents (for review, see [4]). In general, electrophysiology (patch clamp) remains the most accurate technique for analyzing and quantifying the effectiveness of a drug on an ionic conductance. Thus, this approach has been widely used for chloride currents mediated by neuronal GABA_A_ receptors associated with chloride conductance during an inhibitory synaptic transmission. Nevertheless, despite an exceptional fidelity and precision, patch clamp are not suitable for multiple compound screening since this approach is technically demanding, with very low throughput capacity and labor-intensive. Recently, the development of automated electrophysiology has substantially improved the throughput. However, the capability of currently available automated system is not yet compatible with primary screening of large random compounds sets.

Another possibility is the use of fluorescent dyes whose fluorescence intensity is related to the intracellular concentration of ions, an approach widely used for visualizing calcium (for review, [5]). However, this technique has not achieved a general applicability because of several methodological drawbacks, notably for chloride, including sensitivity and specificity, limitations inherent to the loading protocol as well as to photobleaching. In addition, the quantitative determination of chloride fluxes with non-electrophysiological methods amenable to screening approaches has been challenging, in particular because the transmembrane ratio of chloride is low (10∶1) and the equilibrium potential of chloride is generally close to the resting membrane potential of cells, two factors that result in transmembrane fluxes of limited amplitude, raising therefore issues of sensitivity.

Recently, significant progress has been made in Quantitative Phase Microscopy (QPM) techniques [6], [7], [8], [9] that enable to obtain full-field quantitative phase imaging of transparent living cells, allowing to visualize cell structure and dynamics. In contrast to the non-invasive phase contrast (PhC), initially proposed by F. Zernike and by Nomarski's differential interference contrast (DIC), which provides qualitative information about cell structure, QPM, provides a quantitative measurement of the phase shift induced by a transparent specimen on the transmitted wavefront. The phase shift, or the optical path difference (OPD) containing considerable information about the cell morphology as well as intracellular content related to the refractive index properties, can be regarded as a powerful endogenous contrast agent.

The QPM that we have developed, called digital holographic microcopy (DHM), has the ability to explore cell dynamics by providing, from a single recorded hologram, quantitative phase images of living cells with a nanometric axial sensitivity [10]. Practically, an original numerical processing of holograms allows not only to calculate the phase shift but also to reconstruct the whole wavefront diffracted by the specimen [11] and consequently to compensate for aberration [12] and experimental noise (time drift, vibration, defocusing, etc.) thus ensuring a high phase stability making possible to explore biological processes across a wide range of time scales, from milliseconds to hours. Thus, DHM appeared to us as a promising method to monitor ion channel activity because of the capacity of DHM to monitor subtle changes of the intracellular refractive index and cell volume resulting from transmembrane water fluxes associated with the activity of specific ion conductances. We have recently demontsrated this application of DHM to the pharmacological study of glutamatergic ionotropic receptors [13].

In this study, we set out to quantitatively measure from the DHM phase signal the dynamics of chloride currents in response to the modulation of chloride conductances in a well-established biological model used for drug-discovery by High Throughput Screening (HTS). To this aim we have used HEK293 cells, a cell line widely used for pharmacological studies, transfected to express the neuronal ligand-gated channel GABA_A_. The ionic nature of the measured currents was validated by pharmacological analysis as well as by comparing the reversal potential determined by DHM with that measured electrophysiologically with conventional I/V relationships achieved by patch-clamp. Furthermore, the replacement of chloride (Cl^−^) by thiocyanate (SCN^−^) in the extracellular medium amplified the GABA-induced optical signal, permitting the pharmacological characterization the GABA_A_ receptor activity simultaneously in large numbers of unpatched cells. Finally, an original mathematical analysis of the phase response determined by DHM, quantitatively predicts the chloride trans-membrane current, thus providing the possibility to quantitatively measure currents without electrode.

## Results

**Figure 1:**
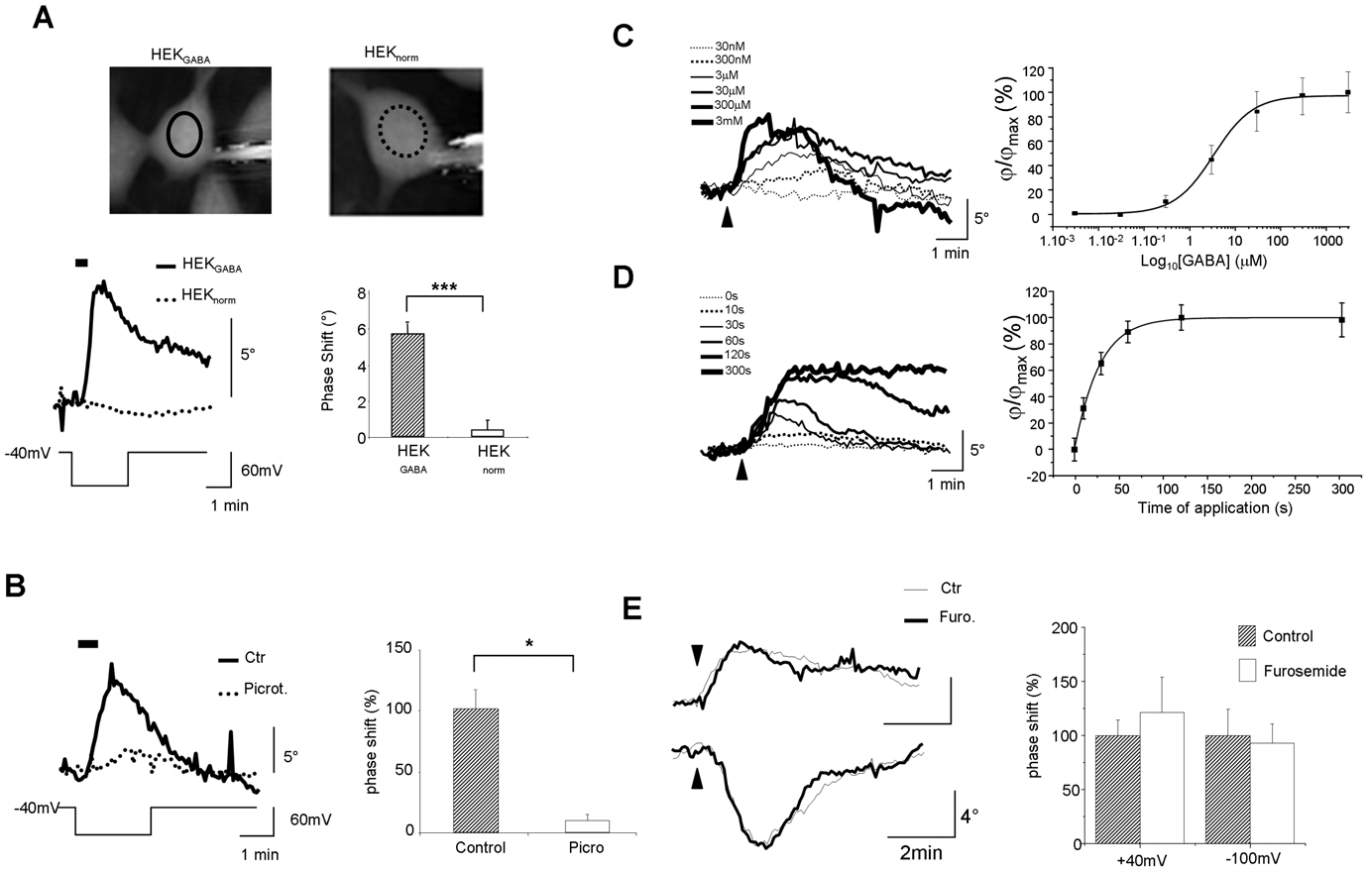
Phase shift is associated with activation of GABA_A_ receptors expressed in HEK_GABA_. **A**: (Top) Phase image of patched HEK_GABA_ (left) and HEK_norm_ (right) recorded by DHM. The full (HEK_GABA_) or the dotted (HEK_norm_) ovals correspond to the region of interest where the phase signal is recorded (scale bar: 5 µm,). (Middle) Application of GABA (3 µM, 30 s; bar) during a pulse of voltage (from −40 mV to −100 mV; 2.5 min) triggered a strong transient increase of the phase signal only in HEK_GABA_. The Bar chart shows the difference between HEK_GABA_ (n = 15) and HEK_norm_ (n = 10) in response to application of GABA at −100 mV (*** p<0.005, unpaired *t*-test). **B**: In presence of picrotoxin (Picrot., 30 µM), application of GABA (3 µM, 30 s; bar) reduces the phase shift (dotted line) (* p<0.05; versus control, paired *t*-test) (n = 10), when compared to control conditions (Ctr; full line). The Bar chart shows the difference between Picrotoxin and Control condition (n = 13) in response to application of GABA (*** p<0.05, paired *t*-test). **C**: (Left) Example of traces of phase shift obtained after the successive application of GABA (from 30 nM to 3 mM, 30 s; arrow head) to the same HEK_GABA_ at −100 mV. With the increase in GABA concentration, the phase shift increased until it reached a plateau. (Right) The graph reports this effect for 6 cells at a holding potential of −100 mV. Fitting the data to the logistic equation yielded an EC_50_ of 3.4 µM. **D**: (Left) Example of traces of phase shift obtained after the successive application of GABA (3 µM, 30 s; arrow head) to the same HEK_GABA_ at −100 mV. With the increase in application time of GABA, the phase shift increased until it reached a plateau. (Right) The graph reports this effect for 9 cells at holding potential of −100 mV. The curve was obtained using a logistic fit with a T _1/2_ of 19.4 s. **E**: (Left) In presence of furosemide (Furo., 100 µM), the phase signal associated to the application of GABA (3 µM, 30 s; arrow head) is not modified (thick line) both, at −100 mV (top) or +40 mV (bottom) when compared to control condition (Ctr, thin line). (Right) The Bar chart shows the absence of difference between furosemide and control condition at +40 mV (n = 5) and −100 mV (n = 4) in response to application of GABA (p>0.05, paired *t*-test).

For all experiments, the transfected HEK cells (HEK_GABA_) cultures had a cell density such that HEK_GABA_ were in contact with neighbouring cells (at least 60% of confluency). Their morphologies were identical to those of the non-transfected HEKs (HEK_norm_) namely polygonal (figure 1A). In terms of electrical properties, HEK_GABA_ had a resting potential of −30.8±0.8 mV (range: −20 to −45 mV; n = 63) and an input resistance of 233±13 MΩ (Range: 150 to 500 MΩ). There were no significant differences in terms of electrical properties between HEK_GABA_ and HEK_norm_ (V_m_: −34.6±1.7 mV; p>0.05; R_inp_: 214±36; p>0.05; n = 10).

### GABA triggers a phase shift in the optical signal on HEK_GABA_

**Figure 2:**
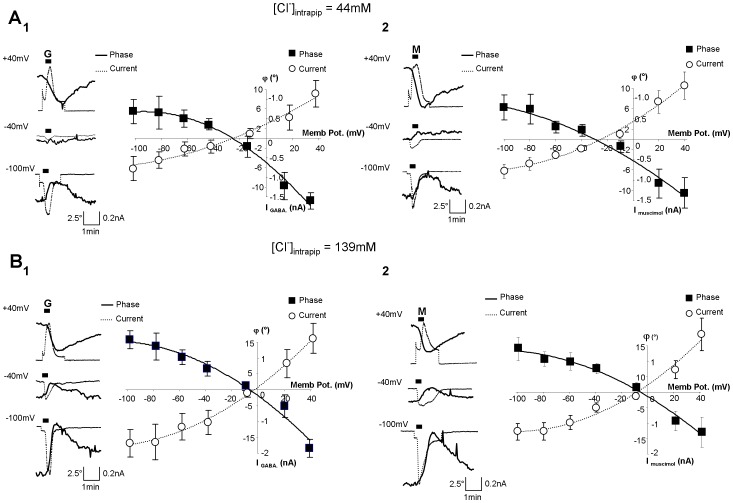
Determination of the value of the reversal potential for Cl from the phase shift evoked by GABA or muscimol application. **A_1_**: (left) Example of 3 simultaneous traces of current (dotted line) and phase shift (thick line) recorded with 44 mM of [Cl^−^]_intrapip_ on the same HEK_GABA_. At −100 mV, application of GABA (3 µM, 30 s) triggered an inward current concomitantly to an increase in the phase signal. Conversely, at +40 mV, same applications of GABA triggered an outward current accompanied by a decrease of the phase signal. Note that for −40 mV (close to the resting potential for Cl), the current and the phase shift were very small. (Right) The φ/V curve (full square and thick line) and the I/V curve (empty circle and thin line) obtained with GABA (n = 7) indicated an E_Cl_ of −26 mV and −26 mV respectively (see also Table 1). **A_2_**: With an application of the GABA_A_ agonist muscimol (1 µM, 30 s, M), the data were similar to those obtained with GABA. With Muscimol, the E_Cl_ was −28 mV with the φ/V curve and −25 mV with the I/V curve (n = 7; see also Table 1). **B_1_** and **B_2_**: (left) In the presence of 139 mM of [Cl^−^]_intrapip_., traces of current and phase shift obtained after application of GABA (3 µM, 30 s; G) (B_1_) or Muscimol (1 µM, 30 s, M) (B_2_) were similar, except for −40 mV, where a larger current and phase shift were detected compared with 40 mM of [Cl^−^]_intrapip_. In this condition, the value of E_Cl_ was shifted to a more positive value. (Right) The φ/V curve (full square and thick line) and the I/V curve (empty circle and thin line) obtained with GABA (n = 6) indicated E_Cl_ of −7 mV and −2 mV, while with muscimol (n = 7), E_Cl_ were −6 mV (φ/V curve) and −8 mV (I/V curve) (see also Table 2).

At −100 mV, bath perfusion of GABA (3 µM, 30 s) on HEK_GABA_ led to a transient increase of phase signal (Δφ = 5.46±1.38°; n = 22), while a similar application of GABA had no effect on HEK_norm_ (Δφ = 0.33±0.55°; n = 6; p<0.005) (figure 1A). In the presence of a GABA_A_ receptor antagonist, picrotoxin (30 µM), the phase response evoked by GABA application was significantly reduced (−82±17%, p<0.05, n = 10) (figure 1B), while the specific agonist, muscimol (1 µM; 30 s, n = 6) mimicked the effect of GABA (figure 2A2). These 1^st^ set of results indicate that the optical signal obtained after application of GABA was associated with the activation of GABA_A_ receptors expressed in HEK_GABA_. Finally, the amplitude of the phase shift depended both on the concentration of GABA (EC_50_ = 3.4 µm) and the length of application (T _1/2_ = 19.4 s) (figures 1C & 1D).

### Determination of the reversal potential of Cl^−^ from the amplitude of the phase shift evoked by GABA application

Through the patch pipette, we have clamped the membrane at various holding potentials. For a given cell, at −100 mV, the application of GABA resulted in a transient increase of phase signal (Δφ = 4.85±2.99°; n = 7) as described above (figure 2A1). In contrast, at +40 mV, GABA triggered a transient strong decrease of phase signal (Δφ = −12.42±3.39; n = 7) (figure 2A1). In reporting the maximal amplitude of the phase shift as a function of the membrane potential, we obtained a relationship which we called “Phase/Voltage” (φ/V), in analogy with the “current/voltage” relationship (I/V) (figure 2A1). In doing so, we were able to detect an outward rectification and also to determine the reversal potential of the ion involved, here Cl^−^ (E_Cl_), in this case −26 mV (figure 2A1; table 1). This value was close not only to the theoretical value of E_Cl_ (E_Cl(Th)_ = −33 mV) calculated with the Nernst equation using values taken from our experimental conditions (table 1), but also to that obtained by classical electrophysiology with the I/V curve (−26 mV; figure 2A1; table 1). At this stage, it is important to not that the large changes in voltage used in this study (from −100 mV to +40 mV), in absence of GABA, were not able to trigger a detectable optical signal by themselves (not shown), reinforcing the fact that the optical signal is only triggered by application of GABA.

**Table 1:** Determination of E_cl_ with a [Cl^−^]_intrapip_ of 44 mM.

[Cl-]_intrapip._ 44 mM				Theo. E_cl_: −33 mV			
GABA			(n = 7)	Muscimol			(n = 7)
	Equation	R^2^	E_Cl_ (mV)		Equation	R^2^	E_Cl_ (mV)
**Phase**	y = −0.0014×2–0.2329×−5.0273	0.9787	−26	**Phase**	y = −0.0015×2–0.4551×−11.368	0.9738	−28
**Current**	y = 4E−05×2+0.0117×+0.2759	0.9767	−26	**Current**	y = 0.0001×2+0.0292×+0.6643	0.9936	−25

To confirm that changes in phase signal were associated with the flow of Cl^−^, we modified the concentration of this anion in the patch pipette ([Cl^−^]_intrapip_.) from 44 mM to 139 mM. Accordingly, the values of the reversal potential for Cl^−^ obtained with the φ/V and I/V relationships were shifted to less negative values (respectively −7 mV and −2 mV; n = 6) and were similar to the value of E_Cl(Th)_ calculated under these new conditions (−4 mV) (figure 2B1; table 2).

**Table 2:** Determination of E_cl_ with a [Cl^−^]_intrapip_ of 139 mM.

[Cl-]_intrapip._ 139 mM				Theo. E_cl_: −4 mV			
GABA			(n = 6)	Muscimol			(n = 7)
	Equation	R^2^	E_Cl_ (mV)		Equation	R^2^	E_Cl_ (mV)
**Phase**	y = −0.0013×2–0.3022×−2.1095	0.9761	−7	**Phase**	y = −0.0022×2–0.5315×−4.3734	0.9738	−6
**Current**	y = 0.0001×2+0.0269×+0.058	0.9958	−2	**Current**	y = 0.0001×2+0.0235×+0.1798	0.9936	−8

Values are means ± SEM. For all conditions, equations, R^2^ and E_cl_ were obtained by using a quadratic polynomial fit (ORIGIN).

Finally, with the specific GABA_A_ receptor agonist, muscimol (1 µM, 30 s), we obtained similar results, the φ/V relationship determining a reversal potential of −28 mV with a [Cl^−^]_intrapip_. of 44 mM (n = 7) (figure 2A2; table 1) while with a [Cl^−^]_intrapip_. of 139 mM (n = 7), the reversal potential was around −6 mV (figure 2; table 2B2), thus confirming that the Cl^−^ flow was due to the opening of the conductance of GABA_A_ receptors.

These results clearly show that the electrochemical properties of a given ionic conductance (here the conductance for Cl^−^) can be determined by DHM with the same precision as that achieved with classical electrophysiological approaches.

### Ionic co-transporters NKCC and KCC are not significantly involved in the genesis of GABA_A_-induced optical signal

It has been established that an alteration in chloride homeostasis linked to a transient but strong activation of GABA_A_ receptors results in an activation of ionic co-transporters KCC and NKCC [14], two membrane proteins co-transporting ions (especially Cl^−^) and water molecules (for review, see [15], [16]), in order to restore a normal chloride homeostasis on both sides of the plasma membrane. To determine the possible involvement of these ionic co-transporters in the genesis of the GABA_A_-induced optical signal, furosemide, a broad spectrum blocker of KCC and NKCC has been used. In presence of this blocker (100 µM), the optical signal induced by application of GABA (3 µM, 30 s) was not significantly modified regardless the membrane potential (−100 mV and +40 mV) (figure 1E), suggesting that these two proteins do not contribute to the optical response mediated by the opening GABA-activated chloride conductance.

### Simultaneous measurement of multiple optical recordings of Cl^−^ flux in unpatched HEK_GABA_ cells

**Figure 3:**
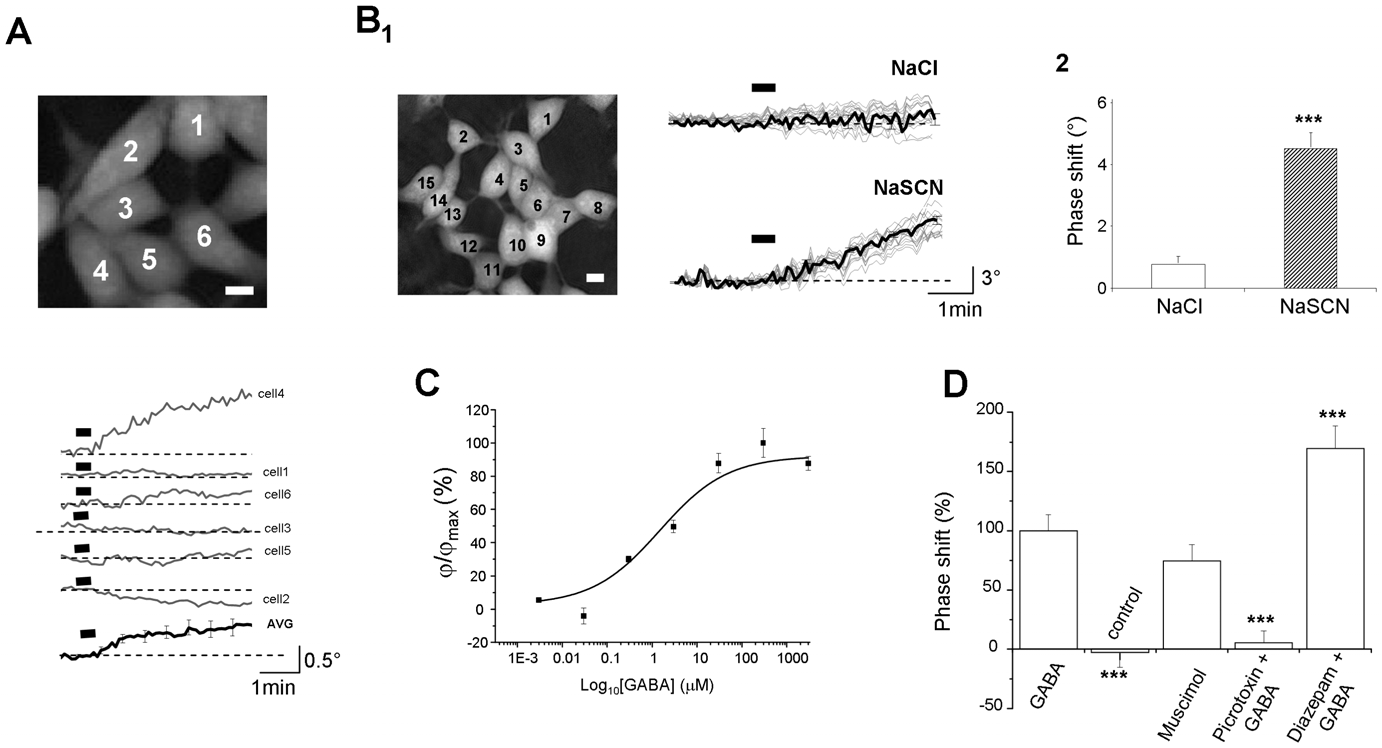
Non invasive multi recording of Cl^−^ flux from several cells. **A**: (top): Phase images of 6 clustered HEK_GABA_ cells visualized in DHM (scale bar: 10 µm). (below): Traces of phase signal (grey line) recorded from corresponding cells showed above. Application of GABA (3 µM, 30 s, bars) triggers an increase in the phase signal for cell n°4 while for cell n°2 a decrease in the optical signal is observed. Note that for cells n° 1, 3, 5 and 6, there are no detectable optical signals. (Bottom) Trace of averaged phase signal (AVG; black line) from the 6 HEK_GABA_ (above). The averaged pick amplitude is less than 0.5°. **B1**: (Left) Phase image of 16 clustered HEK_GABA_ cells visualized in DHM (scale bar: 10 µm). (Right) 2 sets of traces (grey line = individual trace; black line = averaged trace) obtained after application of GABA (3 µM, 30 s, bars) successively in normal (NaCl) and modified (NaSCN) ACSF. **B2**: The Bar chart shows the significant difference between a application of GABA (3 µM, 30 s) in normal (NaCl; n = 188) and Modified (NaSCN; n = 174) ASCF (*** p<0.005, unpaired *t*-test). **C**: The dose-response curve shown was the best fit of the data to the logistic equation described in the Methods section. Fitting the data to the logistic equation yielded an EC_50_ of 1.4 µM. **D**: The Bar chart shows the phase shift response obtained from a large sample of unpatched HEK_GABA_ cells after different types of drugs application: GABA (3 µM, 30 s; n = 178), control (0 µM, 30 s; n = 64), muscimol (1 µM, 30 s; n = 144), Picrotoxin (30 µM)+GABA (3 µM, 30 s) (n = 124) and Diazepam (10 µM)+GABA (3 µM, 30 s) (n = 112). Results are presented in % versus the GABA condition (*** p<0.05, unpaired *t*-test).

The next step consisted in detecting optical signals from unpatched cells in order to explore the possibility of performing simultaneous multiple recordings. As shown in figure 3A, the optical responses triggered by GABA (3 µM, 30 s) from unpatched HEK_GABA_ were heterogeneous resulting either in an increase (25% of recorded cells; n = 47) or a decrease (19%; n = 38) or no detectable changes (56%; n = 105). This result is consistent with the fact that the resting potential of HEK cells (mean value = −31 mV, n = 63) is very close to the reversal potential of Cl^−^ (around-33 mV; table 1), thus explaining that over 50% of the cells exhibited a modest or no detectable phase signal.

To overcome this problem, we altered the Cl^−^ reversal potential by replacing in equimolar amounts most of the extracellular Cl^−^ with SCN^−^ (modified ACSF, see Material and Methods and File SI). In such modified ACSF, application of GABA (3 µM, 30 s), results in a strong positive phase shift simultaneously in almost all HEK_GABA_ cells (up to 85–90% of recorded cells; figure 3B1) corresponding mainly to a Cl^−^ efflux (see File S1 and figure S1). The value of the peak amplitude was significantly higher in modified ACSF (4.44+/−0.59°; n = 174; p<0.005) than in normal ACSF (0.74+/−0.29°; n = 188).

An interesting point is that the EC50 for GABA applied on unpatched HEK_GABA_ perfused with modified ACSF is around 1.4 µM (figure 3C), a value slightly inferior to that determined for patched HEK_GABA_ cells clamped at −100 mV in normal ACSF (3.4 µM; figure 1) but in excellent agreement with the literature [17]. A control application (vehicle solution) had no effect on phase shift (−0.11+/−0.39°; n = 80; figure 3D) demonstrating that the optical effect observed was linked to GABA. Moreover, the pharmacological characteristics of optical responses linked to GABA application (in modified ACSF) were in accordance with those established for GABA_A_ receptors in the literature. Indeed, application of muscimol (1 µM; 30 s) mimicked the effect of GABA (3.31°+/−0.61; n = 144; p = 0.62) while, the phase response evoked by GABA application was totally blocked (0.24+/−0.43°, p<0.005, n = 124) in the presence of picrotoxin (30 µM; figure 3D). Finally, in the presence of diazepam (10 µM), a benzodiazepine known to potentiate the action of GABA on GABA_A_ receptors, the optical response associated with GABA application was significantly increased (6.75+/−0.82°; n = 112; p<0.005; figure 3D) confirming that the effect of GABA is associated with the activation of GABA_A_ receptors.

Taken together these data clearly demonstrate that DHM is a non invasive method (no dye and no electrode) able to characterize pharmacologically the activity of a ligand-gated channel (here the GABA_A_ receptor) simultaneously on a large sample of individual cells. These characteristics could be of great interest for HTS for GABA_A_ receptor/chloride channel modulators.

### The GABA_A_ receptor-mediated current (I_GABA_) can be derived from the phase signal

Our experimental recordings showed that, while the two types of signals (electrical and optical) provide the same information on GABA_A_ receptor properties, their kinetics are strikingly different (figures 2A1 & 4 A1). Thus, at −100 mV, the rise time (τ_rise_) or decay time (τ_decay_) of the phase shift (τ_rise_: 85.0±9.3 s; τ_decay_: 243±20.2 s, n = 8) was significantly longer than for the I_GABA_ (τ_rise_: 23.9±2.3 s; p<0.005; τ_decay_: 47.8±4.7 s; p<0.005). Similarly, at +40 mV, the kinetic constants were also significantly longer for the phase shift (τ_rise_: 92.5±15.3 s; τ_decay_: 220±19.0 s) than for I_GABA_ (τ_rise_: 29.3±1.6 s; p<0.05; τ_decay_: 57.2±1.3 s; p<0.005; n = 6). These measures of kinetics suggest nevertheless a strong relationship between the current and phase signal generated by GABA, where the current is a parameter representing a number of charges per unit of time and the phase signal would be a mirror of the accumulation of these same charges during the total opening time of the conductance.

**Figure 4:**
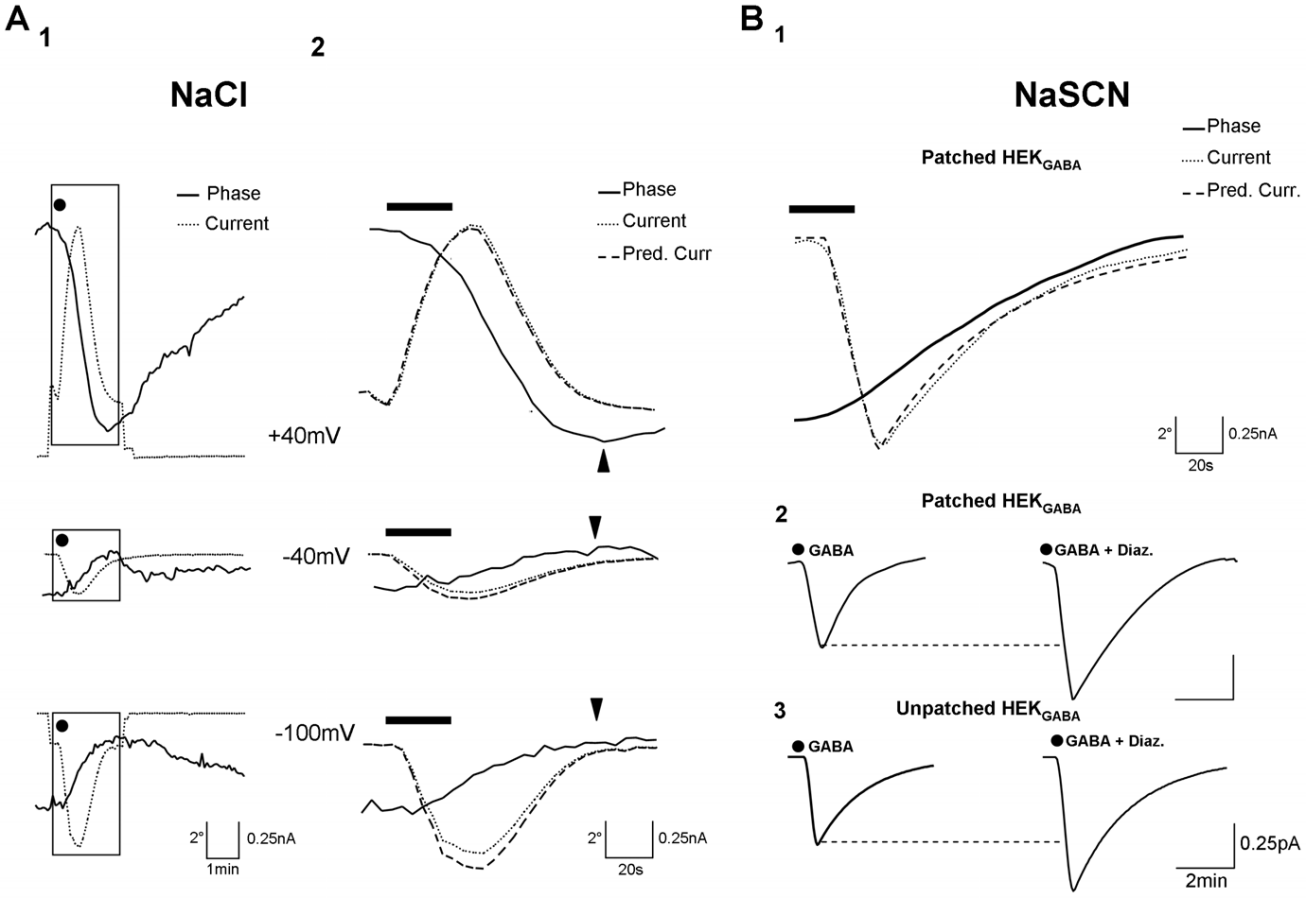
The GABA_A_ gated current can be determined from the phase signal by a simple mathematical relation. **A1**: Simultaneous traces of current (thin line) and phase signal (thick line) obtained after application of GABA (3 µM; 30 s; dot) for 2 different membrane potentials (top: +40 mV; below: −100 mV). Each trace of current and phase signal corresponds to an average of 6 individual current or phase shift from 6 HEK_GABA_ cells. **A2**: Expansion of traces visualized in A (parts defined by rectangles). For each level of membrane potential, the peak of phase shift (indicated by the arrow head) was reached when the I_GABA_ was terminated. According to equation 2 the phase signal can predict the current (Pred. Curr.: dashed line) superimposed to the recorded current. **B1**: Simultaneous traces of current (dotted line) and phase signal (thick line) obtained after application of GABA (3 µM; 30 s; bars) in modified ACSF (NaSCN). Each trace of current and phase signal corresponds to an average of 8 individual current or phase shift from 8 patched HEK_GABA_ and clamped at resting potential (between −25 and −35 mV). According to equation 2 the phase signal can predict the current (Pred. Curr.: dashed line) superimposed to the recorded current. **B2**: Averaged traces of recorded currents from patched HEK_GABA_ after application of GABA alone (3 µM; 30 s; n = 8; left) and GABA+diazepam (10 µM; n = 6; right) in modified ACSF. Note the potentiation of inward current in presence of Diazepam. **B3**: Traces of predicted obtained after derivation by equation 2 of the phase signal from unpatched cells after application of GABA alone (3 µM; 30 s; n = 178; left) and GABA+diazepam (10 µM; n = 112; right) in modified ACSF. In this case also, we retrieved the same magnitude of potentiation with the co-application of GABA and diazepam.

As shown in File S2, this relationship between the current (here I_GABA_) and the DHM phase signal (φ_t_) is explicitly given by equation 2:
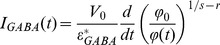
(2)where 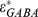 represents the effective volume variations per number of net charges transported through the membrane, which takes into account of any volume variations, including the non-electrogenic ones.

In order to quantitatively derive I_GABA_ from the rapid phase response, several parameters from equation 3 must be known. Practically, ***V_0_*** (initial volume of HEK cells) as well as the parameters **r** (a parameter related to the cell deformation) and **s** (a parameter taking into account transport of substances, including proteins, across cell membranes) have been measured by the decoupling procedure [10], [18]. Consequently, a single parameter remains to be determined, namely 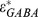, to calculate the current. Practically, as far as the rapid phase is considered, the decoupling procedure has permitted to demonstrate that s value is not statistically different from 1 when HEK cells are considered, indicating that the intracellular refractive index presents a variation mainly resulting from a dilution or a concentration of the intracellular content by ions and water fluxes (see File S2). As far as HEK cells are considered the typical values of the parameter ***r*** are concerned, they are within the range 0.5–0.8, reflecting the fact that the cell deformation associated with the volume change is not isotropic (**r** = 0.33) but preferentially along the z-axis. Practically, figure 4 shows examples of currents derived from the phase signal at three different holding potentials (−100, −40 and +40 mV) as calculated with equation 2. For these calculations, the parameter 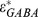 in equation 2, was computed by performing a least squares fit, in order to minimize the sum of the square of the deviations between the measured current from the phase derived current (I_pred._) (figure 4). It must be stressed that the adjustment of this single parameter 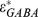 allows obtaining an I_pred._ in good agreement with the measured current. Typical values for the parameters 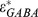 lie within the range of 90–110 µm^3^/nC for HEK cells. With these values of 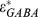, I_pred._ is also in good agreement with the recorded current I_GABA_ when a modified ACSF is considered(Figure 4B1).

Finally, in order to fully take dvantage of the possibility afforded by DHM to optically measure transmembrane currents, we have determined the phase-derived currents corresponding to the mean current over a population of unpatched HEK_GABA_ within the same culture. In modified ACSF condition, equation 2, with parameter 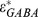 set at values in the range indicated above, provides an I_pred._ from unpatched HEK_GABA_ cells in good agreement with the recorded I_GABA_ from patched cells (figure 4B2). More importantly, we were able to optically measure the potentiation of I_GABA_ by diazepam from unpatched cells. The validity of this optical measure is confirmed by the recording of I_GABA_ potentiation in patched cells (figure 4B2). This stresses the capability of DHM to quantitatively determine characteristic currents of a large cell sample in a non-invasive way (without the use of electrodes or fluorescent dyes), thus making this approach amenable to HTS procedures.

## Discussion

The results reported in this article show that DHM is a simple, reliable and non invasive optical technique for the determination of the pharmacological properties of a chloride conductance simultaneously in a large cell population. Indeed, the optical signal detected with DHM -the quantitative phase response- is an intrinsic one, linked to a physiological process. The illumination system used for DHM is rather conventional (laser diode) and of low power (∼200 µW/cm^2^) [8], [10]. Moreover, with an appropriate analysis of the phase response it is possible to quantitatively obtain the chloride current which generates it, in a strictly non-invasive manner without using recording any electrodes.

The potential offered by DHM to detect and analyse pharmacologically a chloride conductance in a cell population were confirmed by data obtained for GABA_A_ receptors expressed by HEK_GABA_. Practically, the results show that the DHM optical phase responses are specifically linked to the activation of GABA_A_ receptors expressed by HEK_GABA_ (no optical response on HEK_norm_), with pharmacological characteristics in agreement with those already described in the literature [19]. The ionic species underlying the GABA current could be determined by constructing a phase/current plot indicating a reversal potential close to the theoretically-determined equilibrium potential of chloride. It is known that this ligand-gated chloride channel is also permeable to other anions including HCO_3_^−^ [20]. However, one should note that the permeability of HCO_3_^−^ through GABA_A_ receptors is 5 to 10 times lower than that of Cl^−^. Moreover, it is important to note that the optical (and electrical) signals recorded from patched HEK_GABA_ cells have been obtained without HCO_3_^−^ in both bath medium and the intrapipette solution. Consequently, even if we cannot totally rule out the participation of another anion in the GABA-induced optical signal, our results suggest that, in our recording condition, that the optical (and electrical) signals were essentially associated with chloride fluxes. This is particularly interesting, since no reliable optical imaging techniques are available to specifically study Cl^−^ dynamics. To the best of our knowledge this is the first example of an optical technique providing without dye, precise information on specific Cl^−^ fluxes in a quantitative manner.

Another important point concerns the “route” taken by the water molecules accompanying the chloride fluxes. Of course, one route is a passive diffusion through the plasma membrane, a process known to have a slow kinetics. Another possible “route” is a direct water flux through the chloride pore of GABA_A_ receptor. However, it has been shown that chloride conductances are generally poorly permeable to water molecules [20]. Furthermore, the fact that a constant 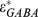 value in equation 2 allows to derive an adequate current response - the early phase of current increase when GABA is applied as well as the delayed phase of the current recovery when GABA is no longer present - is compatible with a low water permeability of chloride conductances. This transport of water during GABA stimulation could be potentially modulated by membrane proteins involved in the transporting of Cl- (and water molecules) such as KCC and NKCC (For review, see [16]). However, our results have clearly shown that there is no contribution of these two ionic co-transporters to the genesis of optical signal.

These results are in accordance with the literature indicating that theses two co-transporters are weakly expressed in native HEK cells [21], [22]. Other proteins transporting Cl^−^, including the HCO_3_^−^/Cl^−^ exchanger [23], voltage-dependent chloride conductances (CIC family) and/or VRAC (Volume Regulatory Anion Channel) [24] could also contribute to the fluxes of Cl^−^ and water triggered by GABA application.. Considering that, in patch clamp recordings, pH was clamped in both compartments (extra- and intracellular medium) it is very likely that the proteins involved in the regulation of pH like HCO_3_^−^/Cl^−^ exchanger are only weakly activated. Concerning a possible involvement of voltage-dependent chloride conductances, large changes in voltage performed in our study (from −100 to +40 mV) without application of GABA did not trigger any detectable phase signal (data not shown), suggesting only marginal, if any, involvement of these chloride conductances in the GABA-induced optical responses. In addition, these type of Chloride channels are weakly expressed in HEK cells [25]. Finally, while one cannot formally exclude a contribution of the VRAC (Volume Regulatory Anion Channel) in the optical signal genesis triggered by GABA, their involvement is very unlikely. Indeed, if VRAC activation were to occur during cell swelling resulting from a massive influx of Cl^−^ (and water molecules), then one would expect a VRAC-dependent signal at different membrane resting potentials during an application of GABA, both when membrane is clamped at +40 mV and at −100 mV. However this can be excluded since a cell shrinkage occurs at −100 mV as a result of Cl^−^ efflux. More importantly, the GABA-induced optical responses, performed on unpatched cell in the NaSCN condition and used to derive currents, correspond to an increase of phase signal i.e. a cell shrinkage, for which a relevant participation of VRAC is very unlikely.

The most important aspect of this imaging technique is the possibility of simultaneous multi-cellular recording which may be very valuable for a possible HTS application, since, in contrast to classical electrophysiological approaches, multiple responses obtained simultaneously from a population of cells can be averaged following the application of pharmacological agents, without disadvantages linked to labelling techniques (loading of dye or contrast agent and/or bleaching). Practically, obtaining such a simultaneous and non-invasive multi-cellular recording, results was achieved through a drastic change of the transmembrane Cl^−^ gradient (substitution of the quasi totality of extracellular Cl^−^ by SCN^−^ in equimolar manner), which allows to obtain values of resting potential (between −20 to −40 mV) that are well separated from the theoretical Cl^−^ reversal potential (in this new condition,  = E_Cl_+30 mV). Within this framework, other approaches changing the transmembrane gradient of Cl^−^ could also be considered such as for example the co-transfection of the GABA_A_ receptor and KCC2, since KCC2 can change the gradient of Cl^−^concentration without affecting the membrane potential [26]. However the conditions used in the experiments reported here are considerably simpler to achieve.

Finally, a simple mathematical expression, with a single unknown parameter “*ε*”, specific for the conductance under study, (here “
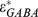
” for GABA_A_ receptor), relating the phase shift to the measured current has been derived and successfully applied to provide a quantitative determination of the ionic current from the DHM optical signal. Practically, the fact that the cell volume is stable before the GABA application i.e 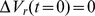 and since a constant value of 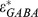 allows to properly calculate the current I_GABA_ from the phase signal, it follows that 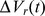 does not significantly contribute to the rapid phase response. Thus the parameter 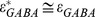 represents the volume variation associated with the net charge movement transported across the pore of GABA_A_ receptor and can thus inform about the membrane permeability to water.

In conclusion, this study describes a novel application of DHM to analyse at the single-cell level non-invasively and without the use of dyes, the optical signature of a specific ionotropic receptor activity as well as its modulation by specific pharmacological agents. In this case the activity of the GABA_A_ receptor selectively permeable to Cl^−^ and its modulation by diazepam, a drug widely used for the management of anxiety disorders in particular are reported. In addition appropriate mathematical treatment of the optical signal affords the possibility to quantitatively determine the dynamics of the current triggered by the GABA_A_ receptor activity, making this technique amenable to use for pharmacological screenings of modulators developed for the management of human pathologies involving dysfunctions of chloride channels.

## Materials and Methods

### Cell preparations

HEK 293 cells stably expressing configurations of rat GABA_A_ receptors (HEK_GABA_) were generously given by Hoffmann-LaRoche (Basel, Switzerland). Briefly, cDNAs encoding rat GABA_A_ α1, β2 and γ2s subunits [27], [28]were subcloned into the expression vectors pIRESpuro2, pIRESneo2 and pIREShygro2 vectors (Clontech, Mountain View, CA), respectively. The pIRES/GABAA α1, β2, γ2s, constructs were sequenced to confirm their nucleotide sequence and then cotransfected into HEK 293 cells at a ratio of 1∶1∶2 (plasmid mass ratio) using the lipofectamine 2000 kit according to the manufacturer's instructions (Invitrogen, Carlsbad, CA, USA). Transfected cells were grown in minimal essential medium (Invitrogen) supplemented with 10% fetal calf serum (Invitrogen), 20 mM HEPES (Invitrogen) and 100 U/ml penicillin/100 µg/ml streptomycin (Invitrogen) for 48 hours and then, the cells were transferred to the selection medium containing 0.3 µg/ml puromycin (Clontech, Mountain View, CA, USA), 300 µg/ml hygromycin B (Roche Diagnostics, Mannheim, Germany) and 200 µg/ml G418 (Invitrogen) for the generation of stable cell lines. Cell colonies were isolated and expression of the GABA_A_ α1β2γ2s receptor was determined by [^3^H]flumazenil binding.

For all experiments, HEK_GABA_ and HEK_norm_ (untransfected) were transferred to a recording chamber and perfused containing in an artificial cerebrospinal fluid (ACSF) containing (in mM): NaCl 140, KCl 3, D-glucose, 5 HEPES 10, CaCl_2_ 3, and MgCl_2_ 2 (pH 7.4; Osm: 290–295 mOsm; Room temperature). For optical multi-recording of GABA_A_ receptor activation, we have substituted in ACSF the NaCl by NaSCN (called modified ACSF) in equimolar manner in order to have less than 5 mOsm between the 2 extracellular solutions. Finally, for some experiments, picrotoxin (30 µM, Tocris) was added to the ACSF. GABA (3 µM, Tocris), Muscimol (1 µM, Tocris) and Diazepam (10 µM, Sigma) were dissolved in ACSF and applied by bath perfusion (for 0 s to 300 s).

### Electrophysiology recording

Whole-cell recordings were made, and signals were amplified by using Multiclamp 700B amplifiers (Axon Instruments, Union City, CA) and digitized by means of an ITC-1600 interface (Instrutech, Great Neck, NY) to a PC computer running Igor Pro (Wavemetrics, Portland, OR). All currents (sampling interval, 5 kHz) were low-pass filtered (2 kHz). They were recorded with pipettes containing (in mM): potassium-gluconate 95, KCl 40, Hepes 10, MgCl_2_ 2 (pH 7.3; Osm: 280–290 mOsm). For some experiments, 95 mM potassium-gluconate was substituted with 95 mM KCl to reach a final concentration of [Cl^−^]_intrapip_. to 139 mM. The pipettes were pulled with a DMZ universal puller.

### Imaging

Digital Holographic Microscopy (DHM) is a full-field interferometric imaging technique that allows to derive from the quantitative phase signal, minute changes in cell refractive index and volume associated with a variety of biological processes without dye or contrast agent (for review, see [29]), in particular when transmembrane water movements are involved [10]. Indeed, changes in ionic permeability are accompanied by a rapid movement of water through the plasma membrane, in particular when involving Cl^−^ [13]. Briefly, as shown by Marquet *et al.* [8], for each pixel of the DHM images, the phase shift induced by the observed cell is given by the following equation:
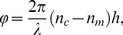
(1)where ***λ*** is the wavelength of the lightsource, ***h*** the cell thickness, ***n_c_*** the mean intracellular refractive index (along the path length corresponding to the thickness *h*), and ***n_m_*** the refractive index of the perfusion solution. Consequently, the phase signal depends on two distinct cell parameters: ***h*** which provides information concerning cell morphology and volume, and ***n_c_*** whose value is related to the amount of non-aqueous material present in the cell and is essentially determined by the protein content [30]. DHM can therefore quantitatively detect small variations of the phase, which mainly depend on the refractive index of the cell ***n_c_*** and cell morphology and volume derived from ***h***. However, the value of the phase is mainly dictated by the intracellular refractive index rather than by cell morphology [10]. In turn, this refractive index is dependent on the protein content of the cell [30], [10]. Accordingly, entry of water will dilute the intracellular protein content resulting in a decrease in the phase while an exit of water will concentrate the protein content leading to an increase in the phase.

The basic design of our imaging system has been described in [10], [31], [13]. Briefly, holograms are acquired with a DHMT 1000 (Lyncée Tech SA, PSE-EPFL). A laser diode produces the coherent light (λ = 683 nm) which is divided by a beam splitter into a reference wave and an object wave. The object wave diffracted by the specimen is collected by a microscope objective and interferes with a reference beam to produce the hologram recorded by the CCD camera. Frequency of hologram acquisition is 0.2 Hz. Reconstruction of the original image from the hologram is numerically achieved by a computer. The reconstruction algorithm provides simultaneous amplitude and quantitative phase images of the cells (Koala software). It is important to note that an extensive quality control of the DHM technique has been published in Rappaz *et al.* [18].

Pratically, before to start a phase recording we waited for a minimum of 10 min in order to obtain a stable baseline. GABA was added after a minimum of 1 min of stable baseline recording for both the optical and the electrical signals.

### Offline analysis

The electrophysiological and optical recordings were analysed by using MATLAB 7.6 (Mathworks Software, Natick, MA) and all curves have been fitted by using ORIGIN 7.5 (Microcal Software, Northampton, MA). GABA concentration-response (or time application-response) profiles were fitted to the following logistic equation: *φ*/*φ*_max_ = 1/[1+(EC50/[GABA])^n^], where *φ* and *φ*_max_ represented the normalized GABA induced phase shift at a given concentration (or time application) and the maximum phase shift induced by a saturating [GABA], EC50 was the half-maximal effective GABA concentration (or time application), and *n* was the slope factor. For both, optical and electrical response of GABA application, rise time (τ_rise_) and decay time (τ_decay_) correspond to 0–100% peak amplitude.

All data are presented as means ± SEM. Student's *t*-test (paired or unpaired) to determine statistical significance (*p*<0.05).

## Supporting Information

Figure S1 **Phase shift changes during application of modified ACSF.** A: 2 representative simultaneous traces of current (thin line) and phase shift (thick line) recorded with 44 mM of [Cl^−^]_intrapip_ (Left; A1) or 139 mM of [Cl^−^]_intrapip_ (Right; A2) after perfusion of a modified ACSF. In both cases, perfusion of such modified ACSF triggered a transient outward current concomitant to a weak transient decrease of phase signal. However, the followed increase of phase signal is speeded up and higher with 139 mM of [Cl^−^]_intrapip_ (A2) corresponding to a stronger inward current. B1: 4 representative simultaneous traces of current (thin line) and phase shift (thick line) recorded with 44 mM of [Cl^−^]_intrapip_ after perfusion of a modified ACSF and application of GABA (3 µM, 30 s, arrow head) at different time (from 30 s to 60 min). We see the speed up of the phase increase for different time intervals between the beginning of the modified ACSF perfusion and GABA application. B2: The graph reports the amplitude of the GABA-induced phase signal (φ_GABA_) for unpatched cells (empty triangle) and patched cells (full square) and I_GABA_ as a function of the interval between the beginning of the modified ACSF perfusion and GABA application. Numbers in the brackets correspond to the number of studied cells.
(TIF)

File S1 **Effects of replacement of nacl with nascn on currents and phase response.**
(DOC)

File S2 **Derivation of the relastionship between phase and current.**
(DOC)
