# Supplementary figures and images for: Simultaneous Optical Recording in Multiple Cells by Digital Holographic Microscopy of Chloride Current Associated to Activation of the Ligand-Gated Chloride Channel GABAA Receptor
Source: PLoS One. 2012 Dec 7;7(12):e51041. doi: 10.1371/journal.pone.0051041 (PMC3517575; doi:10.1371/journal.pone.0051041)

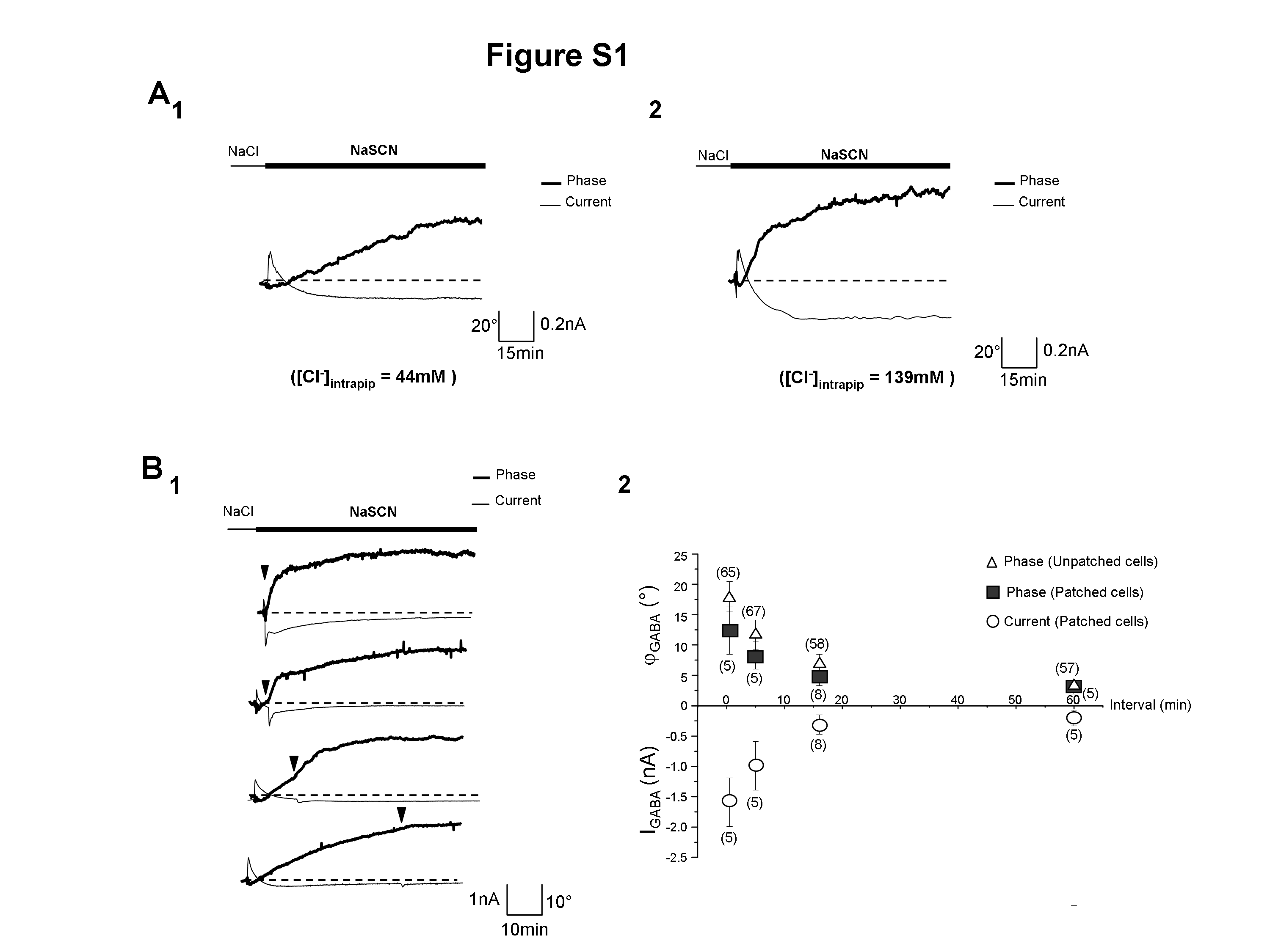

Supplement: Figure S1 — Phase shift changes during application of modified ACSF. A: 2 representative simultaneous traces of current (thin line) and phase shift (thick line) recorded with 44 mM of [Cl−]intrapip (Left; A1) or 139 mM of [Cl−]intrapip (Right; A2) after perfusion of a modified ACSF. In both cases, perfusion of such modified ACSF triggered a transient outward current concomitant to a weak transient decrease of phase signal. However, the followed increase of phase signal is speeded up and higher with 139 mM of [Cl−]intrapip (A2) corresponding to a stronger inward current. B1: 4 representative simultaneous traces of current (thin line) and phase shift (thick line) recorded with 44 mM of [Cl−]intrapip after perfusion of a modified ACSF and application of GABA (3 µM, 30 s, arrow head) at different time (from 30 s to 60 min). We see the speed up of the phase increase for different time intervals between the beginning of the modified ACSF perfusion and GABA application. B2: The graph reports the amplitude of the GABA-induced phase signal (φGABA) for unpatched cells (empty triangle) and patched cells (full square) and IGABA as a function of the interval between the beginning of the modified ACSF perfusion and GABA application. Numbers in the brackets correspond to the number of studied cells. (TIF) [file pone.0051041.s001.tif]
