# Supplementary material for: Simultaneous Optical Recording in Multiple Cells by Digital Holographic Microscopy of Chloride Current Associated to Activation of the Ligand-Gated Chloride Channel GABAA Receptor
Source: PLoS One. 2012 Dec 7;7(12):e51041. doi: 10.1371/journal.pone.0051041 (PMC3517575; doi:10.1371/journal.pone.0051041)
Supplement: File S1 — Effects of replacement of nacl with nascn on currents and phase response. (DOC) [file pone.0051041.s002.doc]

**SUPPLEMENTAL INFORMATION I: EFFECTS OF REPLACEMENT OF NaCl WITH NaSCN ON CURRENTS AND PHASE RESPONSE**

When we patched HEK_GABA_ close to their resting potential (-37.75 +/- 2.3mV; n = 8), replacing NaCl with NaSCN in equimolar concentration (=modified ACSF) typically induces a transient outward current (0.21 +/- 0.06nA; figure S1A) concomitant to an initial transient decrease of the phase signal (-3.2 +/- 1.34°; figure S1A), meaning that this outward current is linked to an entry of anions. This result is fully compatible with the occurrence of a first influx of SCN^-^ (generating the outward current) accompanied by an osmotic transmembrane water influx. Indeed, , during the first minutes of perfusion with the modified ACSF, we created a massive concentration gradient of SCN^-^ due to its strong concentration in the bath medium (140mM) conjugated with its absence in the cell. Finally, after some minutes (less than 10min corresponding to the maximum duration of the outward current), we can assume that the SCN^-^ reached a new chemical gradient in both sides of the plasma membrane.

The transient outward current was followed by a very weak but sustained inward current (-0.04 +-/ 0.06nA; n = 8), associated to a slow increase of the phase signal (; figure S1A). This is consistent with a Cl^-^ efflux driven by a new Cl^-^ equilibrium potential (E_cl_ around +30 mV) far for the resting membrane potential, when NaCl has been replaced with NaSCN.

To validate this working hypothesis, we have changed the intracellular concentration of Cl^-^ (from 44mM to 139mM). In this condition, the initial outward current (0.19 +/- 0.09 nA; p = 0.88) and its associated phase signal decrease (-3.63 +/- 1.34; p = 0.89), occurring early when NaCl has been replaced with NaSCN in bath medium, are not significantly modified. These results support the assumption that these early responses are essentially caused by the concentration gradient of SCN^-^ which is not dependant on the Cl^-^ concentration. On the contrary, the followed inward current is significantly larger even 90min after the beginning of the modified ACSF perfusion (-0.20 +/- 0.09 nA; p < 0.05) (figure S1A)and the phase increase has a larger amplitude. This is consistent with a modification of the Cl^-^ equilibrium potential (E_cl_ ), which induces a larger driving force for the transmembrane Cl^-^ movements when a high internal concentration of Cl^-^(139 mM, E_cl_ around +80 mV) is used. Thus, these results support the assumption that when we substituted Cl^-^ by SCN^-^ in the bath medium, we induce mainly an initial and rapid influx of SCN^-^ followed by a long and slow efflux of Cl^-^.

Coherently, an application of GABA (3μM, 30s) during the slow phase increase, intensifying the process of anion exit (mainly the Cl^-^; figure S1B) by opening of chloride conductance associated to GABA_A_ receptor, speeding up the phase increase. Indeed, application of GABA (3μM, 30s) always corresponds to an inward current associated to a positive phase shift (presumably an exit of Cl^-^) whatever the time interval between the beginning of the modified ACSF perfusion and GABA application (Figure 1SB). Practically, the earlier the GABA application (Figure 1SB), the larger the amplitude of both, electrical and optical responses Consequently, although we cannot entirely exclude a movement of SCN^-^ during the opening of the ligand-gated chloride channel GABA_A_ receptor, the increased optical signal linked to the activation of GABA_A_ receptor seems mainly associated to an exit of Cl^-^ anion. Finally, the fact that the GABA application temporally accelerates the phase increase (after the GABA application, the phase signal resumes its slow increase) reinforces the view of a slow phase increase mainly mediated by Cl^-^ efflux occurs.
