# Supplementary material for: Simultaneous Optical Recording in Multiple Cells by Digital Holographic Microscopy of Chloride Current Associated to Activation of the Ligand-Gated Chloride Channel GABAA Receptor
Source: PLoS One. 2012 Dec 7;7(12):e51041. doi: 10.1371/journal.pone.0051041 (PMC3517575; doi:10.1371/journal.pone.0051041)
Supplement: File S2 — Derivation of the relastionship between phase and current. (DOC) [file pone.0051041.s003.doc]

**SUPPLEMENTAL INFORMATION II: DERIVATION OF THE RELASTIONSHIP BETWEEN PHASE AND CURRENT**

Here we describe how the phase signal changes - reflecting ***V_c_*** variations as well as ***n_c_*** modifications (see equation (1)) - can be used to calculate the associated current in general and I_GABA_ in particular. To address this question we posited two hypotheses. First, we postulated that the ***n_c_*** value depends linearly on the concentration of the different intracellular components. Such an assumption is in agreement with the seminal work of Davies & Wilkins performed within the framework of interference microscopy [1] as well as the recent results concerning the variations of the ***n_c_*** during an hypotonic shock [2]. Second, it is reasonable to assume that the volume variations induced by the water movement accompanying the ionic movements are proportional to the number of ions having passed through the membrane or channels.

By defining the volume factor ***β*** as the ratio between the cell volume measured during GABA application at a time ***t*** and the cell volume (***V_0_***) measured before GABA application, these two hypotheses allow to write:

$n(t)\approx\frac{[C_{0}]}{\beta(t)^{s}}$ (S1.1) $\beta(t)=\frac{\varepsilon_{GABA}\int_{0}^{t} I_{GABA}dt+\Delta V+V_{0}}{V_{0}}$ (S1.2)

where *n(t)* is the refractive index at time t, [**C_0_**] represents the concentration of the different intracellular components before GABA application [mM/ml], *I_GABA_*, the instantaneous net transmembrane current which could be measured by the electrode [C/s] and “***ε_GABA_***” a constant representing the volume variation associated with the net charge movement (here mainly the chloride anion) through the cell membrane [ml/C] in response to the GABA application. In contrast, *ΔV* represents non-electrogenic volume changes corresponding to any cell volume variation except those directly related to a transmembrane net current. Concretely the quantity *ΔV* can include simple electrically neutral ion transport systems as well as more complex processes involved in cell volume regulation. The parameter s has been introduced to take into account cell processes inducing volume variations or not but leading to refractive index changes by mechanisms other than dilution or concentration of the intracellular content by ions and water fluxes. Practically, $s\in[0,1]$ and the case s=1 corresponds to a simple dilution or concentration of the cell content. In contrast, protein release mechanisms involved in the volume regulatory processes modify the refractive index according to equation S1.1 with s<1 knowing that intracellular protein content by itself largely determines the refractive index value .

Finally, according to equations (1), (S1.1), (S1.2) and considering the relation between cell thickness and cell volume variations which can be described as : $\frac{h(t)}{h_{0}}=\beta^{r}(t)$, where *h_0_* is the cell thickness before GABA application and *r* a parameter related to the cell deformation along the z-axis associated with the volume variation, we obtain:

$\left(\frac{\phi_{0}}{\phi(t)}\right)^{1/s-r}=\frac{\varepsilon_{GABA}}{V_{0}}Q_{GABA}(t)+\frac{\Delta V(t)}{V_{0}}+1$ (S1.3a)

with $Q_{GABA}=\int_{0}^{t} I_{GABA}(t)$ and $\phi_{0}$ the phase before the GABA application,

and finally:

(S1.3b)

Thus, equation (S1.3b) establishes a clear relationship between the DHM phase signal (ϕ_t_) and the net transmembrane current (here I_GABA_). It must be pointed out that equation S1.3b can be used to adequately calculate the transmembrane current from the DHM phase shift triggered by the activation of any ionic conductance.

However, for the typical GABA applications considered in this work (no longer than a few tens of seconds), the corresponding phase response can be decomposed into two components, a rapid one accompanying I_GABA_ and a delayed one corresponding generally to a phase recovery while I_GABA_=0. In addition, the two components are separated from each other by a phase plateau behaviour indicating that the possible non-electrogenic volume variations $\Delta V$ can also be decomposed into two components, a rapid ($\Delta V_{r}$) and a delayed ($\Delta V_{d}$) one, which do not overlap and thus are likely to result from different underlying mechanisms. Consequently, only the rapid phase response involving $\Delta V_{r}$will be used to calculate the I_GABA_ and the Equation S1.3b can be rewritten as follows:

(2)

where $\varepsilon_{GABA}^{*}$,formerly defined as , represents the effective volume variations per number of net charges transported through the membrane, which takes into account of any volume variations, including the non-electrogenic ones ($\Delta V_{r}$) .

In order to quantitatively derive I_GABA_ from the rapid phase response, several parameters from equation 2 must be known. Practically, $V_{0}$ as well as the parameters r and s have been measured by the decoupling procedure [2], [3]. Consequently, a single parameter remains to be determined, namely$\varepsilon_{GABA}^{*}$, to calculate the current. Practically, as far as the rapid phase is considered, the decoupling procedure has permitted to demonstrate that s value is not statistically different from 1, indicating that the intracellular refractive index presents a variation directly proportional to the cell volume changes. On the other hand, the typical values of the parameter ***r*** are within the range 0.5-0.8 for HEK cells reflecting the fact that the cell deformation associated with the volume change is not isotropic (r=0.33) but preferentially along the z-axis.

REFERENCES

1. Davies HG, Wilkins MH (1952) Interference microscopy and mass determination. Nature 169: 541.

2. Rappaz B, Marquet P, Cuche E, Emery Y, Depeursinge C, et al. (2005) Measurement of the integral refractive index and dynamic cell morphometry of living cells with digital holographic microscopy. Optics Express 13: 9361-9373.

3. Rappaz B, Barbul A, Emery Y, Korenstein R, Depeursinge C, et al. (2008) Comparative study of human erythrocytes by digital holographic microscopy, confocal microscopy, and impedance volume analyzer. Cytometry Part A 73A: 895-903.
